# PlnTFDB: an integrative plant transcription factor database

**DOI:** 10.1186/1471-2105-8-42

**Published:** 2007-02-07

**Authors:** Diego Mauricio Riaño-Pachón, Slobodan Ruzicic, Ingo Dreyer, Bernd Mueller-Roeber

**Affiliations:** 1 Department of Molecular Biology, Institute for Biochemistry and Biology, University of Potsdam, Karl-Liebknecht-Str. 25 Haus 20, D-14476, Golm, Germany; 2 Cooperative Research Group, Max Planck Institute for Molecular Plant Physiology, Am Muehlenberg 1, D-14476, Golm, Germany

## Abstract

**Background:**

Transcription factors (TFs) are key regulatory proteins that enhance or repress the transcriptional rate of their target genes by binding to specific promoter regions (i.e. *cis*-acting elements) upon activation or de-activation of upstream signaling cascades. TFs thus constitute master control elements of dynamic transcriptional networks. TFs have fundamental roles in almost all biological processes (development, growth and response to environmental factors) and it is assumed that they play immensely important functions in the evolution of species. In plants, TFs have been employed to manipulate various types of metabolic, developmental and stress response pathways. Cross-species comparison and identification of regulatory modules and hence TFs is thought to become increasingly important for the rational design of new plant biomass. Up to now, however, no computational repository is available that provides access to the largely complete sets of transcription factors of sequenced plant genomes.

**Description:**

PlnTFDB is an integrative plant transcription factor database that provides a web interface to access large (close to complete) sets of transcription factors of several plant species, currently encompassing *Arabidopsis thaliana *(thale cress), *Populus trichocarpa *(poplar), *Oryza sativa *(rice), *Chlamydomonas reinhardtii *and *Ostreococcus tauri*. It also provides an access point to its daughter databases of a species-centered representation of transcription factors (OstreoTFDB, ChlamyTFDB, ArabTFDB, PoplarTFDB and RiceTFDB). Information including protein sequences, coding regions, genomic sequences, expressed sequence tags (ESTs), domain architecture and scientific literature is provided for each family.

**Conclusion:**

We have created lists of putatively complete sets of transcription factors and other transcriptional regulators for five plant genomes. They are publicly available through . Further data will be included in the future when the sequences of other plant genomes become available.

## Background

Transcription factors (TFs) are proteins (*trans*-acting factors) that regulate gene expression levels by binding to specific DNA sequences (*cis*-acting elements) in the promoters of target genes, thereby enhancing or repressing their transcriptional rates. The identification and functional characterization of TFs is essential for the reconstruction of transcriptional regulatory networks, which govern major cellular pathways in the response to biotic (e.g. response against pathogens or symbiotic relationships) and abiotic (e.g. light, cold, salt content) stimuli, and intrinsic developmental processes (e.g. growth of organs). Two global types of TFs can be distinguished: basal or general, and regulatory or specific TFs. Basal TFs belong to the minimal set of proteins required for the initiation of transcription (e.g. TATA-box binding protein). Together with RNA polymerase they form the basal transcription apparatus, representing the core of each transcriptional process. In contrast, regulatory TFs bind proximal or distal (up or downstream) of the basal transcription apparatus and act either as constitutive or inducible factors. These proteins influence the initiation of transcription by contacting members of the basal apparatus. Regulatory TFs exert gene-specific and/or tissue-specific functions and influence the transcriptional levels of their target genes in response to different stimuli. In the following when using the term TF, we refer to regulatory TFs.

The large diversity of TFs and *cis- *acting elements they bind to are the source for an enormous combinatorial complexity which allows fine-tuning gene expression control, and gives rise to a huge spectrum of developmental and physiological phenotypes. Therefore, it is not surprising that the manipulation of the expression of TFs often results in drastic phenotypic changes in the organism. This makes them extremely interesting candidates for biotechnological approaches (e.g. [1]). It is widely acknowledged that the evolution of regulatory networks is an important actor in the development of evolutionary novelties, consequently in shaping biological diversity. A deep understanding of transcription factors and their regulatory networks would also improve our understanding of organism diversity [2,3].

The cataloguing of eukaryotic transcription factors started more than a decade ago and has e.g. resulted in the generation of TRANSFAC^®^, a database of *cis*-acting elements and *trans*-acting factors [4]. However, TRANSFAC^® ^includes *A. thaliana *as the only plant species that is extensively represented. Other plant species are covered to a lesser extent (e. g. *Zea mays, Nicotiana tabacum, Lycopersicum esculentum*). Additionally, other TF databases focusing on single plant species are available (for *A. thaliana *[5-7], or *O. sativa *[8]). Kummerfeld and Teichmann [9], have created a server for the prediction of TFs in organisms with sequenced genomes. Up to date, however, none of the currently available databases provides a uniform platform to review plant TF families across several species, encompassing descriptions of each TF family and links to the appropriate literature, and cross-references between the databases by means of orthologous relationships.

Today, nuclear genome sequences are available for several hundreds of organisms, and the sequencing of many more is currently underway. This provides a huge opportunity for making comparisons along different evolutionary branches of the tree of life for various kinds of genes. In this study we have focused on plants and transcription factors. We have predicted the putatively complete sets of transcription factors in five plant species, i.e. the vascular plants *Arabidopsis thaliana *[10], *Populus trichocarpa *[11], *Oryza sativa *[12] and the algae *Chlamydomonas reinhardtii *[13] and *Ostreococcus tauri *[14], and made the data available through a uniform web resource. Currently, various other plant genomes are being sequenced, including genomes from crops and experimental model species (see [15]). Plant Transcription Factor Databases at Uni-Potsdam.de provides an easily usable platform for the incorporation of new TF sequences from these and additional plant species.

## Construction and content

### Source datasets

Sequence data for *A. thaliana *were downloaded from TAIR [16,17], annotation release version 6.0, for *P. trichocarpa *they were downloaded from JGI/DOE [18], annotation release version 1.1, for *O. sativa *from TIGR [19], annotation release version 4.0, for *C. reinhardtii *from JGI/DOE [13], annotation release version 3.1, and for *O. tauri *from the University of Ghent [20], annotation release version August 2006.

### Identification and classification of transcription factors

Transcription factors can be identified and grouped into different families according to their domain architecture, mainly taking into account their DNA-binding domains, as described by Riechmann et al. [21] for *A. thaliana*. We have extended this approach by including new TF families and applied it in a systematic manner to other plant species.

Therefore, in a first step, we identified – using current literature – the list of all domains, which are known to occur in TFs and that are generally employed to classify proteins as transcriptional regulators. The list was established from available PFAM profile Hidden Markov Models (HMMs) (v20.0, [22]), additionally we generated new models for further TF families, as indicated below.

**Figure 1:**
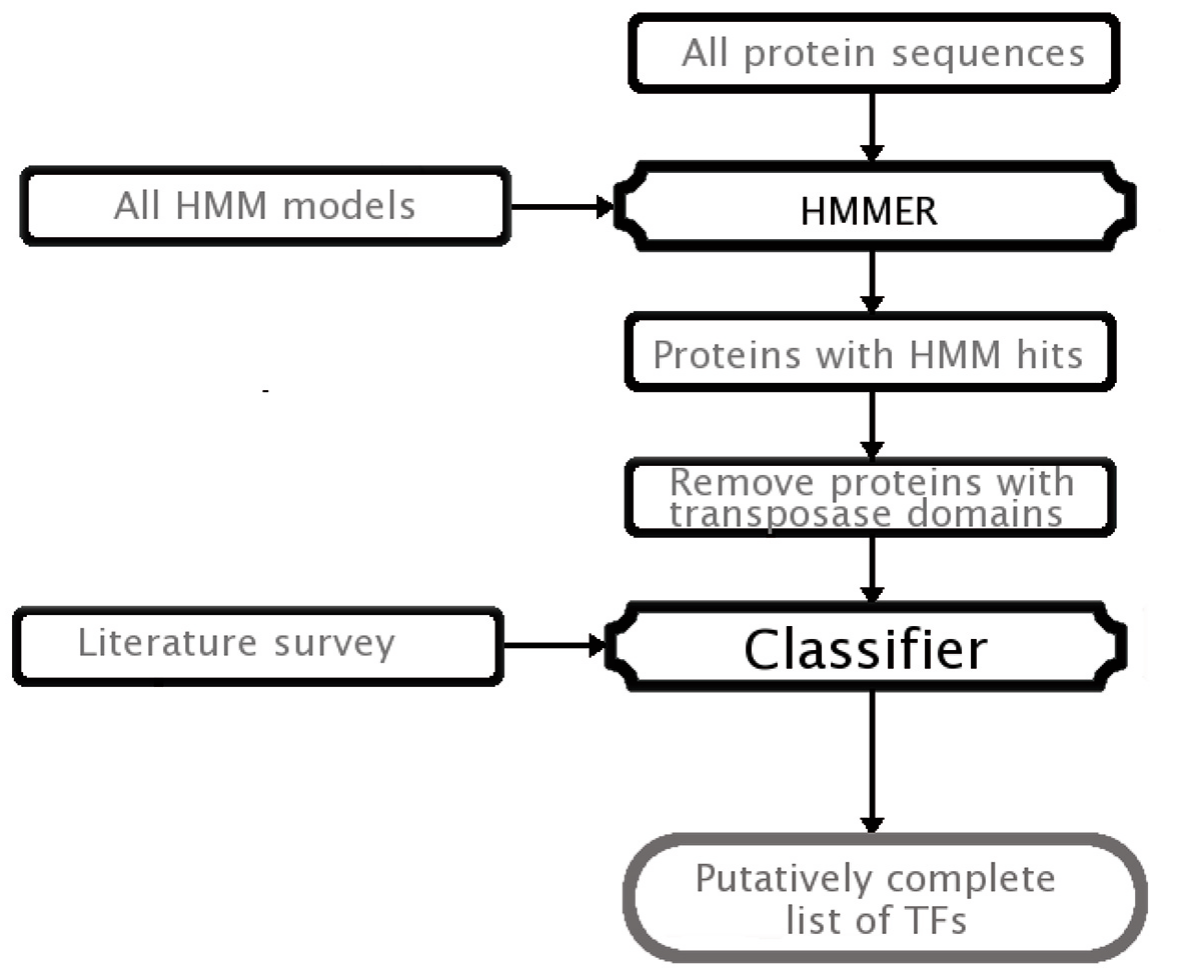
**Pipeline for the identification and classification of TFs**. The pipeline starts with the complete collection of predicted proteins for a given species. Then an HMM search is conducted over this collection keeping all significant hits and discarding all proteins containing a transposase-related domain. Finally the Classifier produces a list of putative TFs grouped into families.

**Figure 2:**
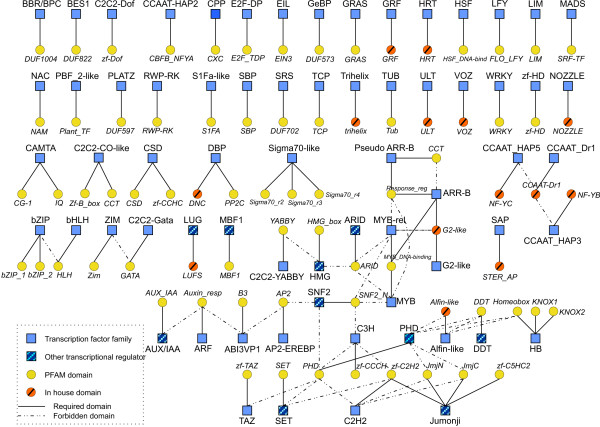
**Rules for the classification of TF families**. Rules for the classification of TFs and other transcriptional regulators depicted as a bipartite graph. Blue squares represent families, TFs are indicated in solid color, other transcription regulators are indicated by shaded squares. Yellow circles represent protein domains from the PFAM database, orange circles represent domains generated in-house. Continuous edges appear when a domain must be present in members of the family. Discontinuous edges indicate that the domain must not appear in members of the family. The profile-HMMs representing the domains Alfin-like and NOZZLE were created based on outputs derived from PSI-BLAST searches at the NCBI protein database; profile-HMMs for the domains CCAAT-Dr1, DNC, G2-like, GRF, HRT, LUFS, NF-YB, NF-YC, STER_AP, trihelix, ULT and VOZ were created from published multiple sequence alignments. All remaining domains were represented by profile-HMMs downloaded from the PFAM database. This figure is accessible via the Plant Transcription Factor Database , and links are provided to the respective TF families and domains.

To group TF proteins into families, we identified – based on previously published data – those domains, or in some cases domain combinations, that were specific for each family ('Literature survey' in Fig. 1). Then, we established a set of rules for each TF family. The rules can be depicted as a bipartite graph with two types of nodes and two types of edges (Fig. 2).

One set of nodes (blue squares) represents protein families (i.e. transcription factors, solid color, or other transcriptional regulators, shaded) and the other set of nodes (yellow circles) represents protein domains. The edges indicate the connections between protein domains and families. A continuous edge represents a required relationship, i.e. the indicated domain must be present in a protein to be assigned to the respective TF family. A discontinuous edge represents a forbidden relationship, i.e. the definition of such a family excludes the presence of the given domain. Rules were implemented in a PERL script as "IF . . . THEN" statements ('Classifier' in Fig. 1).

The general pipeline we have developed for the identification and classification of TFs is shown in Fig. 1. Typically, the process starts with retrieving the complete set of predicted proteins for a given species, followed by a profile-HMM search with all available PFAM HMMs (v20.0, [22]) and the models that we have generated for further TF families. The search is carried out using the software package HMMER (v2.3.2, [23]). All significant HMM hits are kept. For the PFAM models, only those hits with a bit-score larger than the gathering score reported for the HMM were considered significant. For our own HMMs, hits with an e-value smaller than 10^-3 ^and a bit-score threshold that differed for each HMM were considered significant. From this set of significant HMM hits, we discarded all proteins that contained domains having DNA-related activity but not generally regarded as being parts of transcriptional regulators (such as e.g. transposase-related domains). Thereby, we eliminated potential false positives right at the beginning. Finally, we applied the PERL script implementing the set of established rules for the identification and classification of TFs on the remaining set of proteins ('Classifier' in Fig. 1). The script produces as output a list of proteins that belong to the different classes of transcriptional regulators and their classification into the identified families.

For 31 out of 68 families the presence of a single domain was sufficient to assign membership (two out of the 31 families belong to the category of other transcriptional regulators). The remaining families were characterized by combinations of different domains. In this way we were able to classify transcription factors into 58 families plus 10 families for other types of transcriptional regulators, such as chromatin remodeling factors.

Table 1 summarizes the total number of TFs per species identified through the procedure outlined above. We detected 7597 different proteins classified as transcription factors or other transcriptional regulators in the five species analyzed. It is not surprising that the number of TFs generally increases with the number of genes in the genome (e.g. [24]). On average there are 4.2 ± 2.5 TFs per 100 genes. The INPARANOID software implements a variation of the best-reciprocal-BLAST-hits method to search for orthologs between pairs of species [25]. In finding functionally equivalent orthologous proteins INPARANOID has been shown to be the best ortholog identification method [26]. We used INPARANOID to detect orthologs between the analyzed species in a pairwise manner, starting from the complete sets of predicted proteins in each species. The predicted orthologous relationships were used to create cross-references between the species-centered databases.

**Table 1:** Number of TFs per species

Species	Total number of proteins	TFs	TF families	Percentage of TFs
*Ostreococcus tauri*	8236	174 (173)	33	2.1
*Chlamydomonas reinhardtii*	15256	229 (228)	38	1.5
*Arabidopsis thaliana*	30690	2304 (2147)	68	7.5
*Populus trichocarpa*	45555	2723 (2697)	67	6.0
*Oryza sativa*	62827	2516 (2352)	66	4.0

The number of TFs and other transcriptional regulators and the number of different families identified for each of the species studied. Numbers in parenthesis indicate unique protein sequences.

### New HMMs for TF families

For the families Alfin-like, CCAAT-Dr1, CCAAT-HAP3, CCAAT-HAP5, DBP, G2-like, GRF, HRT, LUG, NOZZLE, SAP, Trihelix, ULT and VOZ no appropriated models were found in the PFAM (v20.0) database. Consequently we created our own profile-HMMs based on either published multiple sequence alignments, or on alignments we created based on outputs of PSI-BLAST searches run against the NCBI protein database. The alignments used to build the HMMs are available through our web interfaces.

### Database schemes

**Figure 3:**
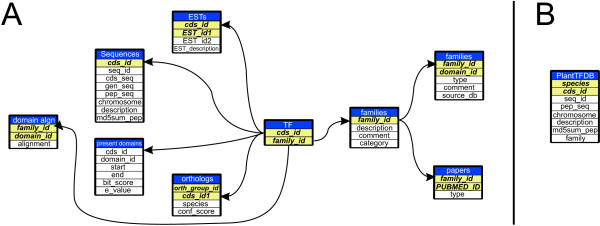
**Database schemes**. Panel A shows the scheme of the species-specific databases. Panel B shows the scheme followed by PlantTFDB. Nine tables structure the information stored in the species-centered databases. **A: **The tables **sequences, present domains, orthologs **and **ESTs **are connected to each other and to the table **TFs **by means of the **cds_id **field. The table **domain_algn **stores the alignments at the domain level for the members of a given family. All five tables contain information about the TFs. The tables **families, relevant domains **and **papers **are connected to each other and to the table **TFs **by means of the field **family_id**. They store the information concerning the TF families. **B**: A single table structures the information for Plant TFDB. Table names appear in blue background, and main keys in green background.

Data of the different TF families are stored in five MySQL relational databases, one for each species, and in a further, global database for PlantTFDB. To uniformly structure the databases two different schemes were implemented (Fig. 3). The first scheme (Fig. 3A) was applied for each of the five independent species-specific databases. The second scheme (Fig. 3B) was implemented for PlantTFDB, which was generated as an entry site to allow access to the species-specific databases.

The basic information in each species-specific database is structured in two sets of tables. One set (right side of the **TF **table) contains in several tables the information about the TF family: literature references, family description and domains relevant for their classification. The field relating the information in these tables is the **family_id**. The second set (left side of **TF **table) contains five tables with the information related to the TFs themselves: sequences, domains present, domain alignments, expressed sequence tags (ESTs), orthologs. The main field here is the **cds_id **that unequivocally identifies every TF. One additional table, the **TF **table relates the two sets of tables. This table has both keys, i.e., **cds_id **and **family_id**, and contains the information about the classification of the transcription factors into families. The PlantTFDB consists of a single table with the following fields: coding sequence identifier, locus identifier, transcription factor family, md5sum of the protein sequence, description of the protein sequence, species name and TF family. The field **md5sum_pep **contains the md5sum of the protein sequence, which is a sequence of 32 hexadecimal digits that identifies unequivocally each protein sequence in the database.

### Web databases

**Figure 4:**
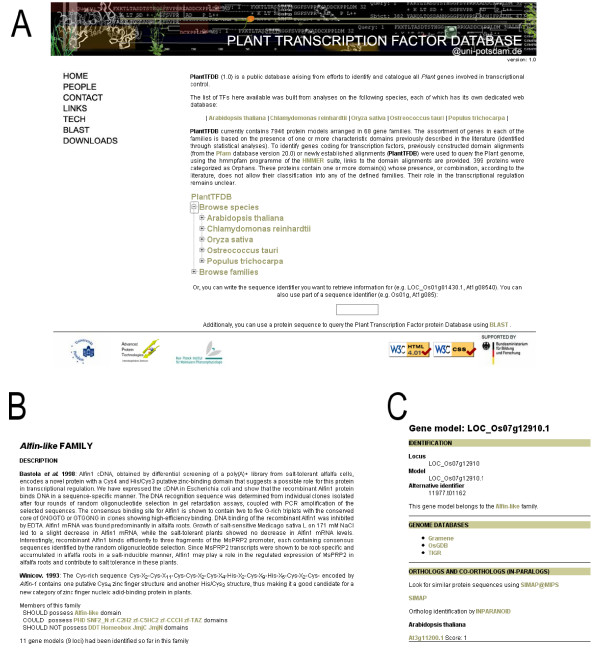
**Web interface**. Panel A shows the starting page for PlantTFDB. The tree menu in the center of the page allows browsing by species or by TF families. Panel B shows part of a typical page for a TF family; a short description and the domains that are important for the definition of the family are shown. Panel C shows part of the page for gene details, which is typical for each member of the DB. Alternative gene names are listed. Links to the genome databases and to the sister TFDBs where orthologs were found are provided.

A web resource with a uniform look-and-feel was developed in PHP (i) for each of the species studied, and (ii) for the PlantTFDB. We have taken care to follow W3 standards regarding HTML v4.01 and CSS v2.1 to assure browser interoperability as much as possible. Data can be downloaded from the databases as plain text files (Fig. 4).

The information provided in the species-specific web databases is linked through the gene identifiers or domain names to different external resources, when available and appropriate: TAIR [17], TIGR's rice genome annotation [19], JGI/DOE's poplar genome [18], and *C. reinhardtii *genome annotation [13], University of Ghent's *O. tauri *genome annotation [20], AthaMap [27], PlantGDB [28], Gramene [29], INPARANOID [30], SIMAP [31], and PFAM [22]. Additional external links to other databases and computational tools will continually be included.

### Quality control

To evaluate the confidence in our lists of putatively complete sets of transcription factors, we decided to compare our predictions to published data sets on detailed phylogenetic single-family analyses in *A. thaliana*. In this way the published analyses were taken as the *gold standard*. We measured the sensitivity and the positive predicive value (PPV) of our approach- in a similar fashion as done by Iida et al. [6] (The terminus 'specificity' used by Iida et al. [6] is in fact the PPV, see [32,33]).

The sensitivity is defined as:

Sensitivity=TPTP+FN,
 MathType@MTEF@5@5@+=feaafiart1ev1aaatCvAUfKttLearuWrP9MDH5MBPbIqV92AaeXatLxBI9gBaebbnrfifHhDYfgasaacH8akY=wiFfYdH8Gipec8Eeeu0xXdbba9frFj0=OqFfea0dXdd9vqai=hGuQ8kuc9pgc9s8qqaq=dirpe0xb9q8qiLsFr0=vr0=vr0dc8meaabaqaciaacaGaaeqabaqabeGadaaakeaacqWGtbWucqWGLbqzcqWGUbGBcqWGZbWCcqWGPbqAcqWG0baDcqWGPbqAcqWG2bGDcqWGPbqAcqWG0baDcqWG5bqEcqGH9aqpdaWcaaqaaiabdsfaujabdcfaqbqaaiabdsfaujabdcfaqjabgUcaRiabdAeagjabd6eaobaacqGGSaalaaa@45AB@

where, *TP *is the number of true positives, i.e. the number of TFs listed in our database that are also found in the gold standard, and *TP + FN*, is the number of true positives plus the number of false negatives, i.e. *TP *+ *FN *is equivalent to the total number of TFs in the gold standard.

The PPV is defined as:

PPV=TPTP+FP,
 MathType@MTEF@5@5@+=feaafiart1ev1aaatCvAUfKttLearuWrP9MDH5MBPbIqV92AaeXatLxBI9gBaebbnrfifHhDYfgasaacH8akY=wiFfYdH8Gipec8Eeeu0xXdbba9frFj0=OqFfea0dXdd9vqai=hGuQ8kuc9pgc9s8qqaq=dirpe0xb9q8qiLsFr0=vr0=vr0dc8meaabaqaciaacaGaaeqabaqabeGadaaakeaacqWGqbaucqWGqbaucqWGwbGvcqGH9aqpdaWcaaqaaiabdsfaujabdcfaqbqaaiabdsfaujabdcfaqjabgUcaRiabdAeagjabdcfaqbaacqGGSaalaaa@39FD@

with the same notation as before, and *FP *being the number of false positives. Thus, *TP *+ *FP *is equivalent to the total number of TFs listed in our database.

According to these definitions, the sensitivity gives an idea of the probability not to miss a true TF: a high sensitivity implies a low number of false negatives. The PPV, in contrast, gives an idea of the goodness of our method at only reporting true TFs: a high PPV implies a low number of false positives. The results of this evaluation are shown in Table 2. For 10 out of 12 tested TF families we obtained sensitivity and PPV values larger than 0.90 for both measurements (bold face in Table 2). Therefore the numbers of false negatives and false positives, respectively, are very low. Thus, the agreement with published results is still acceptable. For the remaining two families the agreement is still reasonable since both values are larger than 0.80, however at least one of them is smaller than 0.90.

**Table 2:** Quality control

Family	Reference	PPV	Sensitivity
**AP2-EREBP**	[39]	146/146 = 1.00	146/147 = 0.99
**ARF**	[40]	21/22 = 0.95	21/23 = 0.91
**AUX/IAA**	[40]	28/28 = 1.00	28/29 = 0.97
bHLH	[41]	122/132 = 0.92	122/154 = 0.80
**bZIP**	[42]	68/70 = 0.97	68/74 = 0.92
**C2C2-Dof**	[43]	35/36 = 0.97	35/36 = 0.97
**C2C2-GATA**	[44]	29/29 = 1.00	29/29 = 1.00
**GRAS**	[45]	32/33 = 0.97	32/33 = 0.97
**MADS**	[46]	99/104 = 0.95	99/108 = 0.92
MYB + MYB-related	[47]	184/209 = 0.88	184/198 = 0.93
**NAC**	[48]	100/101 = 0.99	100/100 = 1.00
**WRKY**	[49]	71/72 = 0.99	71/72 = 0.99

The Positive Predictive Value (PPV) and the Sensitivity were determined for arbitrarily selected *A. thaliana *TF families. For the PPV a deviation from 1.00 means the inclusion of false positives. For the Sensitivity deviations from 1.00 indicate exclusion of true members (false negatives). Families with both values larger than 0.90 appear in bold face.

The computational identification and classification of TFs is a very dynamic process that relies on the available computational models and tools, which in turn rely on the accumulated biological knowledge. This fact is reflected by the calculated Sensitivity and PPV values. As more experimental data become available over time, further improvements in HMMs are expected helping to minimize further the existing gaps between the *gold standards *and the reported data in the database.

## Utility and discussion

Users can start their data-mining either browsing by species, selecting one species and looking at all TF families found in that genome, or browsing by families, selecting one family and looking at the species where this TF family is present. In either case the number of proteins found is shown (see Fig. 4A). When a TF family of interest is located (e.g. Alfin-like family in rice), a click on the name of the family will lead the user to the appropriate species-centered database showing detailed information for that family (see Fig. 4B), where detailed information for each of the protein members can be accessed (e. g. LOC_Os01g66420.1; Fig. 4C). From there the user can navigate to any of the other species for which orthologs have been found. Alternatively, the user can use a preferred protein sequence to search the whole set of TFs in PlnTFDB@Uni-Potsdam, or the species-centered databases, using BLAST.

The availability of all members of a family in several species will facilitate the study of their biological functions, phylogenetic relationships, and the evolution of the DNA-binding domains. For example, Yang *et al*. [34] employed the sequences available in RiceTFDB, which is part of PlnTFDB@uni-potsdam.de, to perform an evolutionary study of DOF TFs from three different species, i.e. Arabidopsis, poplar and rice. Information extracted from our database is currently being used to establish an oligonucleotide-based microarray representing all predicted rice transcription factors (Christophe Perin, CIRAD, Montpellier, personal communication). In our own experiments we recently used the TF sequences listed in RiceTFDB to establish a large-scale quantitative real-time polymerase chain reaction (PCR) platform allowing us to test the expression of more than 2.500 rice TF genes in high throughput (manuscript in preparation). Using this platform we discovered rice TF genes responding to salt and/or drought stress, including, besides others, the genes LOC_Os04g45810 (HB TF), LOC_Os01g68370.3 (ABI3VP1 TF). Notably, the orthologous Arabidopsis genes, i.e. At2g46680.1 and At3g24650, respectively, are known to be affected by salt/drought stress [35,36].

### Future plans and releases

The number of sequenced and annotated plant genomes is rapidly increasing. The computational pipeline described in this article will be applied to new plant genomes as soon as they become available and the new information will be added to future releases of PlnTFDB@uni-potsdam.de. Upcoming versions of the database will also include additional structural data about the domains employed for the identification and classification of TFs, and detailed information about the hierarchical family classification of DNA-binding domains [4,37,38].

We are currently extending the TF discovery pipeline towards large EST collections. The next release of PlnTFDB@uni-potsdam.de will include such information and will classify TFs from plant species whose genomes have not yet been sequenced but for which large EST collections are available.

## Conclusion

We constructed PlnTFDB@uni-potsdam.de, the first database of its kind that provides a centralized putatively complete list of transcription factors and other transcriptional regulators from several plant species. Its daughter databases (OstreoTFDB, ChlamyTFDB, ArabTFB, PoplarTFDB, and RiceTFDB) provide detailed information for individual members of each TF family, including orthologs present in the other species. The latest version of PlantTFDB (vl.O) contains 7597 different protein sequences, grouped into a total of 58 different TF families and 10 additional transcriptional regulator families. The web interface provides access from different starting points, from a gene ID, a protein sequence or a TF family.

## Availability and requirements

All databases can be freely accessed through the WWW using any modern web browser.

PlnTFDB@uni-potsdam.de

**RiceTFDB **

**ArabTFDB **

**PoplarTFDB **

**OstreoTFDB **

**ChlamyTFDB **

## Abbreviations

BLAST, Basic Local Alignment Search Tool. bp, Base pair.

JGI/DOE, Joint Genome Institute/Department of Energy.

NCBI, National Center for Biotechnology Information.

TAIR, The Arabidopsis Information Resource.

TIGR, The Institute for Genomic Research.

## Authors' contributions

BMR, SR and ID participated in the design and coordination of the project. SR and DMRP participated in the definition of the rules for the classification of TFs, and in the design of the web interface. DMRP made all the computational analyses and implemented the web databases. BMR supervised the group as a whole. All authors read and approved the final manuscript.

## References

[B1] Holmes-Davis R, Li G, Jamieson AC, Rebar EJ, Liu Q, Kong Y, Case CC, Gregory PD (2005). Gene regulation in planta by plant-derived engineered zinc finger protein transcription factors. Plant Mol Biol.

[B2] Tautz D (2000). Evolution of transcriptional regulation. Curr Opin Genet Dev.

[B3] Wray GA, Hahn MW, Abouheif E, Balhoff JP, Pizer M, Rockman MV, Romano LA (2003). The evolution of transcriptional regulation in eukaryotes. Mol Biol Evol.

[B4] Matys V, Kel-Margoulis OV, Pricke E, Liebich I, Land S, Barre-Dirrie A, Reuter I, Chekmenev D, Krull M, Hornischer K, Voss N, Stegmaier P, Lewicki-Potapov B, Saxel H, Kel AE, Wingender E (2006). TRANSFAC and its module TRANSCompel: transcriptional gene regulation in eukaryotes. Nucleic Acids Res.

[B5] Guo A, He K, Liu D, Bai S, Gu X, Wei L, Luo J (2005). DATF: a database of Arabidopsis transcription factors. Bioinformatics.

[B6] Iida K, Seki M, Sakurai T, Satou M, Akiyama K, Toyoda T, Konagaya A, Shinozaki K (2005). RARTF: Database and Tools for Complete Sets of Arabidopsis Transcription Factors. DNA Res.

[B7] Davuluri RV, Sun H, Palaniswamy SK, Matthews N, Molina C, Kurtz M, Grotewold E (2003). AGRIS: Arabidopsis gene regulatory information server, an information resource of Arabidopsis cis-regulatory elements and transcription factors. BMC Bioinformatics.

[B8] Gao G, Zhong Y, Guo A, Zhu Q, Tang W, Zheng W, Gu X, Wei L, Luo J (2006). DRTF: a database of rice transcription factors. Bioinformatics.

[B9] Kummerfeld SK, Teichmann SA (2006). DBD: a transcription factor prediction database. Nucleic Acids Res.

[B10] Initiative AG (2000). Analysis of the genome sequence of the flowering plant Arabidopsis thaliana. Nature.

[B11] Tuskan GA, Difazio S, Jansson S, Bohlmann J, Grigoriev I, Hellsten U, Putnam N, Ralph S, Rombauts S, Salamov A, Schein J, Sterck L, Aerts A, Bhalerao RR, Bhalerao RP, Blaudez D, Boerjan W, Brun A, Brunner A, Busov V, Campbell M, Carlson J, Chalot M, Chapman J, Chen GL, Cooper D, Coutinho PM, Couturier J, Covert S, Cronk Q, Cunningham R, Davis J, Degroeve S, Déjardin A, Depamphilis C, Detter J, Dirks B, Dubchak I, Duplessis S, Ehlting J, Ellis B, Gendler K, Goodstein D, Gribskov M, Grimwood J, Groover A, Gunter L, Hamberger B, Heinze B, Helariutta Y, Henrissat B, Holligan D, Holt R, Huang W, Islam-Faridi N, Jones S, Jones-Rhoades M, Jorgensen R, Joshi C, Kangasjärvi J, Karlsson J, Kelleher C, Kirkpatrick R, Kirst M, Kohler A, Kalluri U, Larimer F, Leebens-Mack J, Leplé JC, Locascio P, Lou Y, Lucas S, Martin F, Montanini B, Napoli C, Nelson DR, Nelson C, Nieminen K, Nilsson O, Pereda V, Peter G, Philippe R, Pilate G, Poliakov A, Razumovskaya J, Richardson P, Rinaldi C, Ritland K, Rouzé P, Ryaboy D, Schmutz J, Schrader J, Segerman B, Shin H, Siddiqui A, Sterky F, Terry A, Tsai CJ, Uberbacher E, Unneberg P, Vahala J, Wall K, Wessler S, Yang G, Yin T, Douglas C, Marra M, Sandberg G, de Peer YV, Rokhsar D (2006). The genome of black cottonwood, Populus trichocarpa (Torr. & Gray). Science.

[B12] Project IRGS (2005). The map-based sequence of the rice genome. Nature.

[B13] Chlamydomonas reinhardtii genome annotation – JGI/DOE. http://genome.jgi-psf.org/Chlre3/Chlre3.home.html.

[B14] Derelle E, Ferraz C, Rombauts S, Rouzé P, Worden AZ, Robbens S, Partensky F, Degroeve S, Echeynié S, Cooke R, Saeys Y, Wuyts J, Jabbari K, Bowler C, Panaud O, Piegu B, Ball SG, Ral JP, Bouget FY, Piganeau G, Baets BD, Picard A, Delseny M, Demaille J, de Peer YV, Moreau H (2006). Genome analysis of the smallest free-living eukaryote Ostreococcus tauri unveils many unique features. Proc Natl Acad Sci USA.

[B15] JGI – Sequencing Plans and Progress. http://www.jgi.doe.gov/sequencing/seqplans.html.

[B16] Haas BJ, Wortman JR, Ronning CM, Hannick LI, Smith RK, Maiti R, Chan AP, Yu C, Farzad M, Wu D, White O, Town CD (2005). Complete reannotation of the Arabidopsis genome: methods, tools, protocols and the final release. BMC Biol.

[B17] Rhee SY, Beavis W, Berardini TZ, Chen G, Dixon D, Doyle A, Garcia-Hernandez M, Huala E, Lander G, Montoya M, Miller N, Mueller LA, di SM, Reiser L, Tacklind J, Weems DC, Wu Y, Xu I, Yoo D, Yoon J, Zhang P (2003). The Arabidopsis Information Resource (TAIR): a model organism database providing a centralized, curated gateway to Arabidopsis biology, research materials and community. Nucleic Acids Res.

[B18] Populus trichocarpa genome annotation – JGI/DOE. http://genome.jgi-psf.org/Poptr1_1/Poptr1_1.home.html.

[B19] Yuan Q, Ouyang S, Wang A, Zhu W, Maiti R, Lin H, Hamilton J, Haas B, Sultana R, Cheung F, Wortman J, Buell CR (2005). The institute for genomic research Osal rice genome annotation database. Plant Physiol.

[B20] Ostreococcus tauri genome annotation -Ghent University. http://bioinformatics.psb.ugent.be/genomes/ostreococcus_tauri/.

[B21] Riechmann JL, Heard J, Martin G, Reuber L, Jiang C, Keddie J, Adam L, Pineda O, Ratcliffe OJ, Samaha RR, Creelman R, Pilgrim M, Broun P, Zhang JZ, Ghandehari D, Sherman BK, Yu G (2000). Arabidopsis transcription factors: genome-wide comparative analysis among eukaryotes. Science.

[B22] Finn RD, Mistry J, Schuster-Bockler B, Griffiths-Jones S, Hollich V, Lassmann T, Moxon S, Marshall M, Khanna A, Durbin R, Eddy SR, Sonnhammer ELL, Bateman A (2006). Pfam: clans, web tools and services. Nucleic Acids Res.

[B23] HMMER: profile HMMs for protein sequence analysis. http://hmmer.janelia.org/.

[B24] van Nimwegen E (2003). Scaling laws in the functional content of genomes. Trends Genet.

[B25] Remm M, Storm C, Sonnhammer E (2001). Automatic clustering of orthologs and in-paralogs from pairwise species comparisons. J Mol Biol.

[B26] Hulsen T, Huynen MA, de Vlieg J, Groenen PMA (2006). Benchmarking ortholog identification methods using functional genomics data. Genome Biol.

[B27] Steffens NO, Galuschka C, Schindler M, Bülow L, Hehl R (2004). AthaMap: an online resource for in silico transcription factor binding sites in the Arabidopsis thaliana genome. Nucleic Acids Res.

[B28] Dong Q, Lawrence CJ, Schlueter SD, Wilkerson MD, Kurtz S, Lushbough C, Brendel V (2005). Comparative plant genomics resources at PlantGDB. Plant Physiol.

[B29] Jaiswal P, Ni J, Yap I, Ware D, Spooner W, Youens-Clark K, Ren L, Liang C, Zhao W, Ratnapu K, Faga B, Canaran P, Fogleman M, Hebbard C, Avraham S, Schmidt S, Casstevens TM, Buckler ES, Stein L, McCouch S (2006). Gramene: a bird's eye view of cereal genomes. Nucleic Acids Res.

[B30] O'Brien KP, Remm M, Sonnhammer ELL (2005). Inparanoid: a comprehensive database of eukaryotic orthologs. Nucleic Acids Res.

[B31] Arnold R, Rattei T, Tischler P, Truong MD, Stümpflen V, Mewes W (2005). SIMAP-The similarity matrix of proteins. Bioinformatics.

[B32] Altman DG, Bland JM (1994). Diagnostic tests 1: Sensitivity and specificity. BMJ.

[B33] Altman DG, Bland JM (1994). Diagnostic tests 2: Predictive values. BMJ.

[B34] Yang X, Tuskan GA, Cheng MZM (2006). Divergence of the Dof gene families in poplar, Arabidopsis, and rice suggests multiple modes of gene evolution after duplication. Plant Physiol.

[B35] Söderman E, Mattsson J, Engström P (1996). The Arabidopsis homeobox gene ATHB-7 is induced by water deficit and by abscisic acid. Plant J.

[B36] Nakashima K, Fujita Y, Katsura K, Maruyama K, Narusaka Y, Seki M, Shinozaki K, Yamaguchi-Shinozaki K (2006). Transcriptional regulation of ABI3- and ABA-responsive genes including RD29B and RD29A in seeds, germinating embryos, and seedlings of Arabidopsis. Plant Mol Biol.

[B37] Stegmaier P, Kel AE, Wingender E (2004). Systematic DNA-binding domain classification of transcription factors. Genome Inform.

[B38] Qian Z, Cai YD, Li Y (2006). Automatic transcription factor classifier based on functional domain composition. Biochem Biophys Res Commun.

[B39] Feng JX, Liu D, Pan Y, Gong W, Ma LG, Luo JC, Deng XW, Zhu YX (2005). annotation update via cDNA sequence analysis and comprehensive profiling of developmental, hormonal or environmental responsiveness of the Arabidopsis AP2/EREBP transcription factor gene family. Plant Mol Biol.

[B40] Remington DL, Vision TJ, Guilfoyle TJ, Reed JW (2004). Contrasting modes of diversification in the Aux/IAA and ARF gene families. Plant Physiol.

[B41] Bailey PC, Martin C, Toledo-Ortiz G, Quail PH, Huq E, Heim MA, Jakoby M, Werber M, Weisshaar B (2003). Update on the basic helix-loop-helix transcription factor gene family in Arabidopsis thaliana. Plant Cell.

[B42] Jakoby M, Weisshaar B, Droege-Laser W, Vicente-Carbajosa J, Tiedemann J, Kroj T, Parcy F (2002). bZIP transcription factors in Arabidopsis. Trends in Plant Science.

[B43] Lijavetzky D, Carbonero P, Vicente-Carbajosa J (2003). Genome-wide comparative phylogenetic analysis of the rice and Arabidopsis Dof gene families. BMC Evol Biol.

[B44] Reyes JC, Muro-Pastor MI, Florencio FJ (2004). The GATA family of transcription factors in Arabidopsis and rice. Plant Physiol.

[B45] Bolle C (2004). The role of GRAS proteins in plant signal transduction and development. Planta.

[B46] Parenicová L, de Folter S, Kieffer M, Homer DS, Favalli C, Busscher J, Cook HE, Ingram RM, Kater MM, Davies B, Angenent GC, Colombo L (2003). Molecular and phylogenetic analyses of the complete MADS-box transcription factor family in Arabidopsis: new openings to the MADS world. Plant Cell.

[B47] Yanhui C, Xiaoyuan Y, Kun H, Meihua L, Jigang L, Zhaofeng G, Zhiqiang L, Yunfei Z, Xiaoxiao W, Xiaoming Q, Yunping S, Li Z, Xiaohui D, Jingchu L, Xing-Wang D, Zhangliang C, Hongya G, Li-Jia Q (2006). The MYB transcription factor superfamily of Arabidopsis: expression analysis and phylogenetic comparison with the rice MYB family. Plant Mol Biol.

[B48] Ooka H, Satoh K, Doi K, Nagata T, Otomo Y, Murakami K, Matsubara K, Osato N, Kawai J, Carninci P, Hayashizaki Y, Suzuki K, Kojima K, Takahara Y, Yamamoto K, Kikuchi S (2003). Comprehensive Analysis of NAG Family Genes in Oryza sativa and Arabidopsis thaliana. DNA Res.

[B49] Ulker B, Somssich IE (2004). WRKY transcription factors: from DNA binding towards biological function. Curr Opin Plant Biol.

